# Identifying complications of interventional procedures from UK routine healthcare databases: a systematic search for methods using clinical codes

**DOI:** 10.1186/1471-2288-14-126

**Published:** 2014-11-28

**Authors:** Kim Keltie, Helen Cole, Mick Arber, Hannah Patrick, John Powell, Bruce Campbell, Andrew Sims

**Affiliations:** Newcastle upon Tyne Hospitals NHS Foundation Trust, Newcastle Upon Tyne, UK; Institute of Cellular Medicine, Newcastle University, Kragujevac, UK; York Health Economics Consortium, York, UK; National Institute for Health and Care Excellence, London, UK

**Keywords:** Adverse effects, Medical errors, Patient safety, Health information systems

## Abstract

**Background:**

Several authors have developed and applied methods to routine data sets to identify the nature and rate of complications following interventional procedures. But, to date, there has been no systematic search for such methods. The objective of this article was to find, classify and appraise published methods, based on analysis of clinical codes, which used routine healthcare databases in a United Kingdom setting to identify complications resulting from interventional procedures.

**Methods:**

A literature search strategy was developed to identify published studies that referred, in the title or abstract, to the name or acronym of a known routine healthcare database and to complications from procedures or devices. The following data sources were searched in February and March 2013: Cochrane Methods Register, Conference Proceedings Citation Index – Science, Econlit, EMBASE, Health Management Information Consortium, Health Technology Assessment database, MathSciNet, MEDLINE, MEDLINE in-process, OAIster, OpenGrey, Science Citation Index Expanded and ScienceDirect. Of the eligible papers, those which reported methods using clinical coding were classified and summarised in tabular form using the following headings: routine healthcare database; medical speciality; method for identifying complications; length of follow-up; method of recording comorbidity. The benefits and limitations of each approach were assessed.

**Results:**

From 3688 papers identified from the literature search, 44 reported the use of clinical codes to identify complications, from which four distinct methods were identified: 1) searching the index admission for specified clinical codes, 2) searching a sequence of admissions for specified clinical codes, 3) searching for specified clinical codes for complications from procedures and devices within the International Classification of Diseases 10^th^ revision (ICD-10) coding scheme which is the methodology recommended by NHS Classification Service, and 4) conducting manual clinical review of diagnostic and procedure codes.

**Conclusions:**

The four distinct methods identifying complication from codified data offer great potential in generating new evidence on the quality and safety of new procedures using routine data. However the most robust method, using the methodology recommended by the NHS Classification Service, was the least frequently used, highlighting that much valuable observational data is being ignored.

**Electronic supplementary material:**

The online version of this article (doi:10.1186/1471-2288-14-126) contains supplementary material, which is available to authorized users.

## Background

Complications are an important consideration in assessing the safety and efficacy of new interventional procedures [1, 2], but reliable data are difficult to acquire. Complications of interventional procedures may relate to the procedures themselves, devices used to conduct the procedure, or to implanted devices. In recent times, the complications of devices have become a particular concern, with the high profile examples of breast implants [3] and metal-on-metal hip implants [4].

For assessing effectiveness in clinical practice, observational research can complement evidence from randomised controlled trials, by identifying the nature and frequency of adverse events [5] and detecting rare outcomes [6–8]. For interventional procedures, a common approach is to establish a dedicated clinical register to capture information about diseases, procedures and devices in selected study populations [9], but there can be many obstacles to establishing dedicated registers and submission to them may be incomplete [10]. An alternative is to use routine healthcare databases [11] that capture information from large populations across a broad range of interventions. Such data are available more readily and at a lower cost than bespoke patient registers [12, 13] and have been used to assess outcomes of interventional procedures in clinical practice [14, 15].

Several authors have developed and applied methods to routine data sets to identify the nature and rate of complications following interventional procedures. But, to date, there has been no systematic search of such methods. The aim of this study was to find, classify and appraise published methods which used routine healthcare databases to identify complications from interventional procedures, in a United Kingdom (UK) setting. This paper considers methods based on analysis of clinical codes.

## Methods

This study was based on a systematic search of scientific literature and did not involve primary research with human subjects, human material or human data; no ethical approval was required.

### Data sources

The following databases were searched: Cochrane Methodology Register, Conference Proceedings Citation Index – Science, Econlit, EMBASE, Health Management Information Consortium, Health Technology Assessment database, MathSciNet, MEDLINE, MEDLINE in-process, OAIster, OpenGrey, Science Citation Index Expanded and ScienceDirect. Where database functionality allowed, searches were limited to results published from 1987 and in English language. Searches were carried out in February and March 2013. The MEDLINE search strategy is described in Additional file 1. This was adapted as appropriate for each database searched.

### Study selection

Six healthcare databases used in the UK, identified from an initial scoping search, were considered in this study. Two were routine administrative databases used to record episodes of patient care in the UK National Health Service (NHS): Hospital Episode Statistics (HES) [16] and the Clinical Practice Research Datalink (CPRD) [17]. Two were databases used to record deaths: the Office for National Statistics (ONS) database [18] and the Primary Care Mortality Database (PCMD) [19]. The remaining two were adverse incident databases: the National Reporting and Learning System (NRLS) [20] and the Medicines and Healthcare products Regulatory Agency (MHRA) database of adverse drug reactions, defective medicines, device failures and blood product safety reports. Synonymous and related terms for these data sources included the General Practice Research Database (GPRD) (former name for CPRD), the National Patient Safety Agency (NPSA) which operated the NRLS, and Datix™, a commercial product used by many hospitals to manage incidents and which provides summary reports to NRLS.

Papers reporting primary or secondary research with a clearly defined methodology were included if they described the use of at least one of the data sources included in the scope with the intention of identifying complications from procedures or devices. Exclusion criteria were: non-English language studies, conference abstracts, studies published before 1987 (when both HES and GPRD administrative databases were established) and studies with methods not considered repeatable from the description provided.

Titles and abstracts of the literature search results were independently reviewed by two authors (KK and HC) and arbitrated by a third (AS). When a paper could not be ruled out from the information available in the title and abstract, the full paper was retrieved. Two authors (KK and HC), arbitrated by a third (AS), independently reviewed the full papers and applied the eligibility criteria to each. Studies from eligible papers were inductively classified (KK and AS arbitrated by HC) into different methods of identifying complications; each method being a combination of type of database and whether direct clinical coding of complications or surrogate indicators of complications were used. Of the eligible papers, those which reported methods using clinical coding were reviewed, and data extracted using a standard template.

## Results and discussion

### Eligible papers

**Figure 1:**
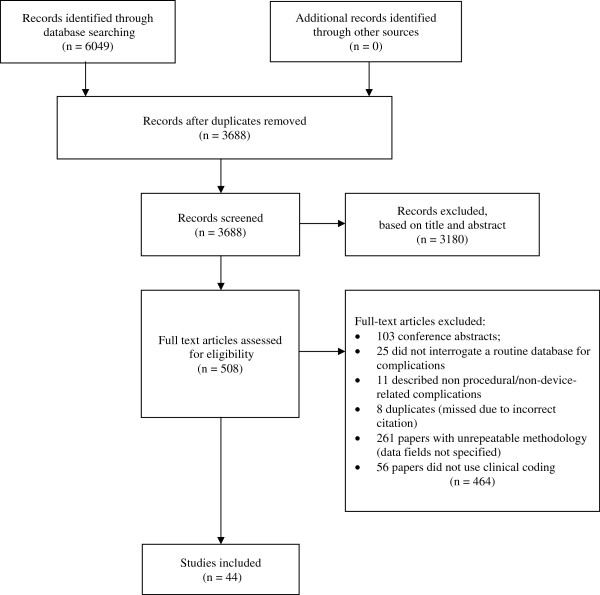
**Flow of information through different phases of the study** [21] **.**

In total, 3688 records were assessed for relevance by two authors (arbitration by a third was required for 43 papers, 1.2%); 3180 were subsequently excluded using information from the title and abstract. Full text articles were retrieved for the remaining 508 records. Of these, 408 did not meet the eligibility criteria, leaving 100 full articles for further analysis (Figure 1). Forty-four articles reported the identification of complications using methods based on clinical codes (59.1% of which also used surrogate measures to identify complications) and were included in this study (a summary of which is included in Additional file 2). The remaining sixty-six articles were excluded on the basis of using only surrogate measures to identify complications.

### Characteristics of eligible studies

Table 1 shows the distribution of studies by medical specialty and routine healthcare database. The clinical coding schemes used in these databases were the International Classification of Diseases (ICD) [22], the Office of Population Census and Surveys Classifications of Interventions and Procedures (OPCS) [23] and READ [24] codes.

**Table 1:** **Distribution of eligible studies by medical specialty and routine healthcare database**

Medical specialty	Routine healthcare database			Total no. of papers
	HES	CPRD	SMR ^†^	
Gastro-intestinal	16	1		17
Orthopaedic	9			9
Vascular	5			5
All medical specialties	3			3
Urology	3			3
Obstetrics & gynaecology	2		1	3
Respiratory	3			3
Radiology		1		1
*Total studies*	41	2	1	

^†^Indicates a database which was not included within literature search terms but was detected incidentally from review of full articles.

Thirty-seven articles (94.1%) included a period of follow-up which extended beyond the duration of the initial (*i.e.* index) admission; this ranged from 2 days to 23 years in the studies found. Twenty papers captured co-morbidities for each patient; 13 used the Charlson comorbidity index [25], 5 recorded only specified comorbidities and 2 used the modified Royal College of Surgeons Charlson comorbidity index [26]. Socioeconomic deprivation was captured in 9 studies; 8 used the Carstairs Index of Deprivation [27] and one used the Index of Multiple Deprivation, 2004 [28].

Four distinct methodological approaches (referred to hereafter as approaches (a-d)) based on clinical coding were found during inductive classification of the forty-four articles, these identified complications by: a) searching the index admission for specified clinical codes, b) searching a sequence of admissions for specified clinical codes, c) applying the methodology recommended by NHS Classification Service, and d) conducting manual clinical review of diagnostic and procedure codes.

#### Complications identified by searching the index admission for specified clinical codes (a)

In 13 papers, the authors began by listing codes for potential peri-procedural complications or emergency corrective procedures and then searched for them in the index admission only [29–41]. All used the HES database in which procedures are coded using the Office of Population Censuses and Surveys Classification of Interventions and Procedures (OPCS-4) system and diagnoses are coded using the International Classification of Diseases 10th revision (ICD-10) system.

Ten of these studies searched for diagnostic codes [30–32, 34–40]. Of these, six [30–32, 37, 39, 40] included clinical codes specifically intended for recording complications. These were the ICD-10 diagnosis codes from the range T80-T88 (“complications of surgical and medical care, not elsewhere classified”), or equivalent values from earlier versions of ICD classification. For example, El-Dhuwaib *et al.*[31] compared outcomes of laparoscopic versus open repair of inguinal hernia, and searched for the presence of, amongst others, code T81.2 (“accidental puncture and laceration during a procedure, not elsewhere classified”), to indicate the occurrence of a procedural complication. However, some studies used unqualified disease codes to indicate a complication [30–32, 34, 39, 40]. For example, Holt *et al.*[39] counted the presence of ICD-10 code I63 (“cerebral infarction”) as a complication of aortic aneurysm repair.

Only two studies restricted their use of diagnostic codes to those specifically intended for recording complications [32, 34] to overcome the difficulty of discriminating between complications and co-morbidities. For example, the Department of Health searched for a specific complication code related to wound site infection for all types of admission in England over one year [32].

Eight of the 13 studies [29, 30, 32–35, 38, 41] considered the occurrence of certain additional procedures during the index admission to indicate a complication. For example, Almoudaris *et al.*[29] studied colorectal cancer resections and the frequency of reoperations (including washout of abdomen and drainage of abscesses). Three studies [30, 34, 38] used a combination of specific diagnoses and additional procedures to identify complications. For example, Onwere *et al.*[34] captured, with high sensitivity, incidences of blood transfusion associated with caesarean section deliveries in women with placenta praevia through the presence of particular OPCS-4 codes and ICD-10 diagnosis codes.

#### Complications identified by searching a sequence of admissions for specified clinical codes (b)

In an extension of the technique described above (a), the authors of 35 papers listed codes for potential complications and emergency corrective procedures and then searched for them in a sequence of admissions. Thirty-three of these used HES and two used CPRD. Subsequent admissions were used to identify complications from the index admission that became apparent after discharge.

Twenty-one of the 35 papers in this category searched readmission records [29, 31, 32, 42–59], with follow-up intervals that ranged from within 2 days [31] to 13 years [58]. For example, Kulkarni et al. [52] identified postoperative complications of total hip and knee replacement in patients who also had bariatric surgery by searching for medical complications, such as myocardial infarction, occurring within 30 days of surgery, deep vein thrombosis or pulmonary embolism within 90 days, hip revisions within 12 months, or dislocations within 18 months.

Ten of the 35 papers searched both the index admission and readmissions [30, 36, 60–67]. For example, Hilton & Cromwell [61] estimated rates of fistula in hysterectomy patients by searching for specific diagnostic codes within the index admission and readmissions within 12 months.

The remaining four papers of the 35 in this category searched admissions before, during and after the index admission [68–71]. Admissions before the index admission were used to help identify co-morbidities and to distinguish them from complications. For example, Mamidanna *et al.*[70] identified medical complications of emergency colorectal surgery in the elderly by capturing specified diagnoses (*e.g.* angina, atrial fibrillation) during the index admission or in readmissions within 30 days, only if they were not recorded during previous admissions in the five years preceding surgery. A similar, but alternative approach was adopted by Smith *et al.*[71], who used HES to determine whether there was an increased risk of cancer in the early years after metal-on-metal hip replacement due to biological effects of the metals.

#### Complications identified using the methodology recommended by NHS Classification Service (c)

The ICD-10 coding scheme includes provision for the clinical coding of complications from procedures and devices. There are also disease codes which indicate a complication in their own right (*e.g.* I97 – “post-procedural disorders of the circulatory system, not elsewhere classified”) and there are codes which can be used to qualify other diseases as being iatrogenic. For example, if code Y83 (“surgical operation and other surgical procedures as the cause of abnormal reaction of a patient, or of later complication, without a mention of misadventure at the time of the procedure”) followed, or qualified, code I63 (“cerebral infarction”), it would indicate that the stroke was the result of a procedure.

Only one paper made full use of this convention to identify adverse events. Aylin *et al.*[72] searched HES for the presence of 41 different ICD-10 three character codes, including codes for iatrogenic disease and qualifying codes, specifically intended to identify the frequency of ‘adverse events’ and ‘medical or surgical misadventure’. They applied this technique to HES records of all inpatient admissions in NHS in England between 1999 and 2003, and estimated that, on average, 2.2% of all episodes include a code for an adverse event, and 0.03% for a misadventure.

#### Complications identified by manual clinical review of diagnostic and procedure codes (d)

Manual clinical review of HES records was reported in three papers [53, 56, 66]. For example, Cathcart *et al.*[56] manually examined all diagnostic and procedure codes for circumcisions, when the length of stay exceeded one day or when a patient was readmitted within 30 days.

## Conclusions

### Statement of principal findings

The aim of this study was to find, classify and appraise published methods based on the analysis of clinical codes from routine healthcare databases in a UK setting to identify complications from interventional procedures. Four distinct methods of identifying complications were found, and to our knowledge, this is the first search and classification of published methods for identifying complications from routine data.

Coding schemes provide a compact, structured and logically consistent scheme for representing complex clinical records. They provide the basis for the development of algorithms to identify procedures, diseases and events of interest. Several authors have applied algorithms to large codified data sets, such HES, to identify complication rates at national level for procedures of interest, but each of these approaches has limitations in overcoming particular challenges. For example, searching only for a prospective list of diagnoses (deemed as complications) may lead to underestimation of the true peri-procedural complication rate if complications were not anticipated from earlier trial outcomes or expert opinion. Additionally, diagnosis codes (*e.g.* I63 – “cerebral infarction”) are used to code both comorbidities and complications, and therefore searching for them in isolation may overestimate the peri-procedural complication rate. Manual review of codified data by clinical experts, in the absence of patient notes, is a very time-consuming and impractical alternative approach which does not overcome either of these limitations and it may also be subject to operator error or observer bias.

Using the presence of multiple procedure codes occurring within the same hospital admission as a marker of procedural complication may also be misleading because there are many reasons why additional procedures may be necessary. Without clinical review or access to case notes, additional procedures cannot be confidently used as a surrogate for complications. In addition, searching only for a prospective list of possible repair procedures may miss complications that were resolved by other means and may lead to an underestimation of the true complication rate.

HES data have been used most frequently for assessing short-term complications (typically those arising peri-procedurally and within 30 days). Their utility for identifying longer-term complications remains uncertain for two main reasons. Firstly, the process of constructing comprehensive longitudinal patient records from multiple data sources (*e.g.* primary and secondary care) is complex and laborious. Secondly, over extended periods of time, the certainty with which adverse outcomes can be attributed to particular procedures is reduced. None of the eligible studies that considered longer-term outcomes has fully overcome these limitations.

Only one paper included in our study used the facility implicit within the ICD-10 coding scheme to identify short-term complications. This method, which is based on the use of supplementary codes to indicate complication and specific codes for iatrogenic disease, overcomes the problem of separating complications from co-morbidities and avoids the need to use the incidence of additional procedures as a surrogate marker of complication. It is surprising that it has been applied so rarely to date.

### Strengths and weaknesses of the study

This study was based on a systematic search for published studies that identified complications from specific routine data sources using analysis of clinical codes. Because the search found so many eligible studies, we were able to group together methods which had been applied to diverse clinical disciplines and appraise the strengths, limitations and clinical utility of each.

Although the study was limited to routine databases used in the United Kingdom, the identified methods are more widely applicable. Methods using the international standard, ICD-10, for coding diseases are directly applicable to other healthcare settings and an increasing number of countries are using ICD for the purposes of reimbursement and healthcare resource allocation [22]. For example, Quan et al. recently surveyed 31 countries, all of which use ICD coding systems, and found that 24 (77.4%) specifically used ICD-10 [73]. OPCS and READ coding schemes are specific to the UK setting, but the strengths and weaknesses of the methods that used these schemes for identifying complications are applicable to any databases using codified clinical terms.

Because the scope was intentionally non-specific to a particular clinical context the search strategy was designed to find studies which included non-specific complication related index and free text terms. Studies that used specific terms to describe complications would not necessarily have been retrieved but may have included generalizable methods which were not otherwise captured.

### Meaning of the study: possible implications for clinicians and policymakers

The ICD-10 scheme includes provision for the clinical coding of complications and adverse incidents. Using codes in the ranges T80-88 and Y40-84 to distinguish comorbidities from complications is recommended strategy for the NHS [74]. However, few studies to date have taken full advantage of the capabilities of ICD-10 for this purpose. But why has that been the case? It may be speculated that full exploitation of these codes requires relatively sophisticated manipulation of code sequences at episode-level (for example to group a main disease code with a qualifying Y-code immediately following it) and that these techniques have not been readily available to most researchers.

The next version of the International Classification of Diseases, ICD-11 [75], will include an extensive revision of ICD-10 chapters 19 and 20 which are concerned with healthcare related injury. ICD-11 will provide a mechanism to encode the cause of harm, the mode or mechanism of harm and the harm incurred. Specific consideration is also being given to the number of diagnosis fields needed to capture safety events [76], improved methods for recording morbidity and for reporting the timing of diagnoses [73].

The improvements proposed for ICD-11 are an important development for clinicians, clinical coders and researchers, but our study has shown that information about healthcare related injury, already recorded in health records using ICD-10, remains largely unexploited. Important evidence that would likely change current practice is being ignored and more should be done to use it for patient benefit.

### Unanswered questions and further research

Further research is needed into the utility of clinical codes used to indicate iatrogenic disease. In particular there is a need to develop algorithms that exploit the use of such codes and to validate these algorithms using corroborative datasets, such as bespoke clinical registers or local patient records.

Complications with longer term manifestations require data linkage with mortality records, incident databases and other routine national databases in order to provide a longitudinal patient record, but there is a need for the development and assessment of new methods, based on such data linkage, to capture longer-term events and consequences reliably.

## Authors’ information

HP and JP are Consultant Clinical Advisers to the National Institute for Health and Care Excellence (NICE) Interventional Procedures Programme. BC is Chair of the NICE Interventional Procedures Advisory Committee and the NICE Medical Technologies Advisory Committee. KK, HC, and AS are employed by the Newcastle upon Tyne NHS Hospitals Foundation Trust and MA is employed by the York Health Economics Consortium, which are partner organisations that provide an External Assessment Centre to NICE.

## Electronic supplementary material

Additional file 1: **Literature search strategy.** Literature search strategy terms. (DOCX 15 KB)

Additional file 2: **Summary of eligible studies describing methods of deriving complications using codified data from routine healthcare databases.** Large (A3 landscape) table. (DOCX 20 KB)

## Abbreviations

CPRD: Clinical practice research datalink
FTR-S: Failure to rescue surgical
GPRD: General practice research database
HES: Hospital episode statistics
ICD: International classification of diseases
LoS: Length of stay
MHRA: Medicines & healthcare products regulatory agency
NHS: National health service
NPSA: National patient safety agency
NRLS: National reporting & learning system
ONS: Office for national statistics
OPCS: Office of population censuses & surveys classification of interventions & procedures
PCMD: Primary care mortality database
UK: United Kingdom.
