# Supplementary material for: Identifying complications of interventional procedures from UK routine healthcare databases: a systematic search for methods using clinical codes
Source: BMC Med Res Methodol. 2014 Nov 28;14:126. doi: 10.1186/1471-2288-14-126 (PMC4280749; doi:10.1186/1471-2288-14-126)
Supplement: Supplementary file 1 — Additional file 1: Literature search strategy. Literature search strategy terms. (DOCX 15 KB) [file 12874_2014_1142_MOESM1_ESM.docx]

Additional File 1: Literature search strategy.

Strategy initially developed for Ovid MEDLINE(R) In-Process & Other Non-Indexed Citations and Ovid MEDLINE(R) 1946 to Present. Search date 28/02/13. This strategy was adapted as appropriate for each additional database searched.

1. Hospital Episode$ Statistic$.ti,ab. 411
2. HES.ti,ab. 3553
3. General Practic$ Research Database$.ti,ab. 922
4. GPRD.ti,ab. 318
5. Clinical Practic$ Research Data$.ti,ab. 6
6. CPRD.ti,ab. 19
7. "National Reporting and Learning System$".ti,ab. 23
8. NRLS.ti,ab. 43
9. National Patient Safety Agency$.ti,ab. 175
10. NPSA.ti,ab. 94
11. Datix$.ti,ab. 4
12. "Medicines and Healthcare products Regulatory Agency$".ti,ab. 526
13. MHRA.ti,ab. 517
14. Office for National Statistics.ti,ab. 396
15. ONS.ti,ab. 1024
16. Primary Care Mortality Database$.ti,ab. 0
17. PCMD.ti,ab. 8
18. or/1-17 6944
19. (ae or co).fs. 2611541
20. complication$1.ti,ab. 546570
21. Product Surveillance, Postmarketing/ 5266
22. exp Postoperative Complications/ 391852
23. exp Intraoperative Complications/ 34258
24. ((adverse or undesirable or harm$ or serious or critical) adj3 (effect$1 or affect$1 or incident$1 or reaction$1 or event$1 or outcome$1)).ti,ab. 250341
25. (safety or side effect$1 or side affect$1).ti,ab. 389542
26. (re-attend$ or reattend$).ti,ab. 270
27. (re-admit$ or readmit$ or re-admission$ or readmission$).ti,ab. 11747
28. (re-operat$ or reoperat$ or surgical revision$ or repeat surg$).ti,ab. 28403
29. patient readmission/ 6860
30. reoperation/ 61528
31. or/19-30 3468372
32. 18 and 31 2334
33. limit 32 to (english language and yr="1987 -Current") 2134
