# Supplementary material for: Identifying complications of interventional procedures from UK routine healthcare databases: a systematic search for methods using clinical codes
Source: BMC Med Res Methodol. 2014 Nov 28;14:126. doi: 10.1186/1471-2288-14-126 (PMC4280749; doi:10.1186/1471-2288-14-126)
Supplement: Supplementary file 2 — Additional file 2: Summary of eligible studies describing methods of deriving complications using codified data from routine healthcare databases. Large (A3 landscape) table. (DOCX 20 KB) [file 12874_2014_1142_MOESM2_ESM.docx]

Additional File 2: Summary of eligible studies describing methods of deriving complications using codified data from routine healthcare databases

Definitions: Clinical Practice Research Datalink (CPRD), Hospital Episode Statistics (HES), Medical Defence Unit (MDU), Medicines and Healthcare products Regulatory Agency (MHRA), Medical Protection Services (MPS), National Health Service Litigation Authority (NHSLA), National Reporting and Learning System (NRLS), Office of National Statistics (ONS), Scottish Morbidity records (SMR).

| Author  (Year) | Data source | Medical specialty | Description of methodology to derive procedural complications  (length of follow up) | **Subcategory** | Comorbidity/ Socio-economic status  recorded |
| --- | --- | --- | --- | --- | --- |
| Almoudaris (2011) [32] | HES | GI | Specified return to theatre (index admission)  Failure to Rescue-Surgical (FTR-S) rate (index admission) | **a** | Yes |
| Almoudaris (2013) [28] | HES | GI | Specified reoperation (28 day)  Failure to rescue-surgical (FTR-S) rate (index admission) | **a, b** | Yes |
| Amar (2012) [42] | CPRD | Radiology | Occurrence of named diagnoses (length of study) | **b** | Yes |
| Aylin (2004) [71] | HES | All specialties | Occurrence of general diagnoses categories (length of study) | **c** | No |
| Ballal (2009) [34] | HES | GI | Occurrence of specified procedures indicating conversion (index admission) | **a** | No |
| Burns (2011) [48] | HES | GI | Occurrence of named diagnoses (28 day) | **b** | Yes |
| Burns (2011) [62] | HES | GI | Occurrence of specified diagnoses and procedures indicating reoperation (index admission, 28 day) | **b** | Yes |
| Burns (2013) [29] | HES | GI | Specified return to theatre (index admission, 28 day) | **a, b** | Yes |
| Cathcart (2006) [55] | HES | Urology | All diagnoses and procedures analyses retrospectively to identify complications (index admission, 30 day)  Occurrence of specified procedures indicating reoperation (6 month) | **b, d** | No |
| Clarke (2004) [40] | HES | Respiratory medicine | Occurrence of specified procedure (index admission) | **a** | No |
| Cooper (2011) [49] | SMR | Obstetrics & Gynaecology | Occurrence of specifies diagnoses and procedure indicating recurrence (length of study) | **b** | Yes |
| David (2008) [53] | HES | GI | Occurrence of specified diagnoses and procedures indicating reoperations (length of study) | **b** | No |
| David (2009) [35] | HES | GI | Occurrence of specified procedures indicating reversal (length of study)  Occurrence of specified diagnoses (index admission) | **a, b** | Yes |
| Dixon (2004) [57] | HES | Orthopaedics | Occurrence of specified diagnoses and procedure indicating revision (length of study) | **b** | No |
| Dyer (2013) [41] | HES | Urology | Occurrence of specified diagnoses (1 year) | **b** | No |
| El-Dhuwaib (2012) [43] | HES | GI | Occurrence of specified diagnosis and procedure indicating repair (length of study) | **b** | No |
| El-Dhuwaib (2013) [30] | HES | GI | Occurrence of specified diagnoses (index admission)  Emergency readmission combined with occurrence of specified diagnoses (2 days, 30 days) | **a, b** | No |
| Hilton (2012) [60] | HES | Obstetrics & Gynaecology | Occurrence of specified diagnoses and procedures (index admission, 1 year) | **b** | No |
| Holt (2007) [38] | HES | Vascular | Occurrence of specified diagnoses (index admission) | **a** | No |
| Holt (2007) [39] | HES | Vascular | Occurrence of specified diagnoses (index admission) | **a** | No |
| Jameson (2010) [64] | HES | Orthopaedics | Occurrence of specified diagnoses (90 day)  Occurrence of specified procedures indicating repair (30 day) | **b** | No |
| Jameson (2012) [44] | HES | Orthopaedics | Occurrence of specified diagnoses (30 day, 90 day) | **b** | Yes |
| Jameson (2013) [58] | HES | Orthopaedics | Occurrence of specified procedure indicating repair (30 day, 18 month)  Occurrence of specified diagnoses (30 day, 90 day) | **b** | Yes |
| Jen (2008) [36] | HES | Orthopaedics | Occurrence of specified diagnoses (index admission) | **a** | Yes |
| Judge (2006) [56] | HES | Orthopaedics | Occurrence of specified diagnoses and procedures indicating revision (5 year) | **b** | Yes |
| Judge (2007) [66] | HES | Urology | Occurrence of specified diagnoses (index admission, 30 day)  Occurrence of specified procedure indicating secondary procedure (1 year) | **b** | Yes |
| Kim (2011) [50] | HES | Respiratory medicine | Occurrence of specified procedures indicating repair (length of study) | **b** | No |
| Kulkarni (2011) [51] | HES | GI | Occurrence of specified diagnoses and procedures indicating reoperation (30 day) | **b** | No |
| Lalmohamed (2012) [45] | CPRD | GI | Occurrence of specified diagnoses (length of study) | **b** | Yes |
| Mamidanna (2012) [67] | HES | GI | Occurrence of specified diagnoses (index admission, 30 day)  Occurrence of specified procedures indicating reoperation (index admission, 30 day) | **b** | Yes |
| Mamidanna (2012) [68] | HES | GI | Return to the operating theatre for specified procedures (index admission, 28 day)  Occurrence of specified diagnosis (1 year) | **b** | Yes |
| Mamidanna (2012) [69] | HES | GI | Occurrence of specified diagnoses (30 day) taking into account comorbidities.  Occurrence of procedures indicating repair (index admission, 28 day) | **b** | Yes |
| Moxey (2011) [63] | HES | Vascular | Occurrence of specified diagnoses (1 year) | **b** | Yes |
| Moxey (2012) [61] | HES | Vascular | Occurrence of procedures indicating revision/repair (index admission, 1 year) | **b** | Yes |
| National Prospective Tonsillectomy Audit (2007) [65] | HES | Respiratory Medicine | Occurrence of specified diagnoses and procedures (index admission, 28 day)  All diagnoses and procedures analyses retrospectively to identify complications (index admission, length of study) | **b, d** | No |
| NHS Executive (1997) [31] | HES | All specialties | Occurrence of named diagnoses wound infection (index admission)  Occurrence of procedure indicating disease recurrence (1 year) | **a, b** | No |
| Onwere (2011) [33] | HES | Obstetrics & Gynaecology | Occurrence of specified diagnoses and procedures (index admission) | **a** | No |
| Raleigh (2008) [37] | HES | All specialties | Occurrence of specified diagnoses (length of study) | **a** | No |
| Sibanda (2008) [54] | HES | Orthopaedics | Revisions (1 year, 3 year) | **b** | No |
| Sinha (2013) [59] | HES | GI | Occurrence of specified procedures indicating repair (1 year) | **b** | Yes |
| Smith (2012) [70] | HES | Orthopaedics | Occurrence of specified diagnoses (length of study) | **b** | No |
| Stafford (2012) [46] | HES | Orthopaedics | Revisions (length of study) | **b** | Yes |
| Sutton (2012) [47] | HES | Vascular | Occurrence of specified diagnoses (1 year) | **b** | No |
| West (2010) [52] | HES | GI | Bleeding (7 day)  All diagnoses and procedures analyses retrospectively to identify deaths associated with procedure (7 day) | **b, d** | No |
